# Supplementary material for: Effectiveness of Abdominal Ultrasonography for Improving the Prognosis of Pancreatic Cancer during Medical Checkup: A Single Center Retrospective Analysis
Source: Diagnostics (Basel). 2022 Nov 23;12(12):2913. doi: 10.3390/diagnostics12122913 (PMC9777348; doi:10.3390/diagnostics12122913)
Supplement: Supplementary file 1 [file diagnostics-12-02913-s001.zip › diagnostics-1936861-supplementary.pdf]

Table S1. Patients with PC diagnosed based on other approaches (group 8).

CT for abnormal serum level of liver enzyme	1
CT in medical checkup	2
Abdominal ultrasonography for abnormal liver enzyme	4
High serum level of CEA	2
EGD for anemia	1
EGD in medical checkup	1

CT: computed tomography, CEA: carcinoembryonic antigen, EGD : esophagogastroduodenoscopy

Table S2. Details of patients in group 7.

	Age	M/F	PS	Place where PC was found	Comorb-idities	Specialty of clinic doctor	Subspecialty of clinic doctor	Tumor location	Tumor size (cm)	Ultrasonographic findings				PDAC / IPMN derived carcinoma	Stage of cancer	Thera-py	Prognosis
					DM, HT, HL					Tumor in pancreas	MPD dilatation	Cyst in pancreas	Tumor in liver				
1	67	F	0	Medical checkup center	none			body	20	●				PDAC	1	Ope	Died after 1764 days
2	70	F	0	Clinic	none	Internal medicine	Gastro-enterology	body	20	●				PDAC	2a	Ope	No relapse after 2690 days
3	72	F	0	Clinic	HL	Internal medicine	Unknown	groove	35	●				PDAC	2a	Ope	No relapse after 2619 days
4	65	M	0	Medical checkup center	HL			head	20		●			PDAC	2b	Ope	No relapse after 2621 days
5	72	M	0	Referral center	HT, HL			body	08	●				PDAC	2	Chem	Died after 633 days

6	59	M	0	Medical checkup center	none	Internal medicine	Respiratory medicine	tail	52				●	PDAC	4	Chem	Died after 294 days
7	69	M	0	Clinic	HT	Internal medicine	Gastro-enterology	body	12		●			PDAC	4	Chem	Died after 362 days
8	80	F	0	Clinic	DM, HL	Internal medicine	Gastro-enterology	uncus	30		●	●		PDAC	4	Chem	Changed hospital after 172 days
9	78	F	0	Clinic	DM, HL	Internal medicine	Gastro-enterology	head ~ body	42	●	●			PDAC	2b	Ope	Died after 586 days
10	80	F	0	Clinic	HT, HL	Internal medicine	Gastro-enterology	head ~ body	0		●			PDAC	0	Ope	Alive with relapse after 1756 days
11	64	F	0	Clinic	HL	Internal medicine	Gastro-enterology	body	5		●			IPMN-derived carcinoma	0	Ope	No relapse after 1195 days
12	84	F	0	Clinic	HT, HL	Internal medicine	Gastro-enterology	uncus	14		●			PDAC	2a	Ope	No relapse after 992 days

13	84	F	0	Clinic	none	Internal medicine	Gastro-enterology	body	27		●			PDAC	2a	Ope	No relapse after 834 days
14	86	M	0	Clinic	HT, HL	Internal medicine	Gastro-enterology	tail	50		●			PDAC	4	Chem	No relapse after 677 days
15	75	M	0	Clinic	DM, HT	Internal medicine	Gastro-enterology	body	10	●				PDAC	2b	Ope	No relapse after 642 days
16	78	M	0	Referral center	HT, HL			tail	44				●	PDAC	4	Chem	Dead after 134 days
17	75	M	0	Clinic	HT, HL	Internal medicine	Gastro-enterology	head	15		●			PDAC	2b	Ope	No relapse after 539 days

Group 7 included the patients with pancreatic cancer (PC) who were diagnosed through abdominal ultrasonography during medical checkup of asymptomatic individuals.

M: male, F: female, PS: performance status, DM: diabetes mellitus, HT: hypertension, HL: hyperlipidemia, US: abdominal ultrasonography, ●: present, MPD: main pancreatic duct, PDAC: pancreatic ductal adenocarcinoma, IPMN: intraductal papillary mucinous neoplasm, Ope: operation, Chem: chemotherapy
